# Maxwell Equations without a Polarization Field, Using a Paradigm from Biophysics

**DOI:** 10.3390/e23020172

**Published:** 2021-01-30

**Authors:** Robert S. Eisenberg

**Affiliations:** 1 Department of Applied Mathematics, Illinois Institute of Technology, Chicago, IL 60616, USA; Reisenberg@iit.edu; Tel.: +1-708-932-2597; 2 Department of Physiology and Biophysics, Rush University Medical Center, Chicago, IL 60612, USA

**Keywords:** polarization, maxwell equations, gating current, dielectric constant

## Abstract

When forces are applied to matter, the distribution of mass changes. Similarly, when an electric field is applied to matter with charge, the distribution of charge changes. The change in the distribution of charge (when a local electric field is applied) might in general be called the induced charge. When the change in charge is simply related to the applied local electric field, the polarization field **P** is widely used to describe the induced charge. This approach does not allow electrical measurements (in themselves) to determine the structure of the polarization fields. Many polarization fields will produce the same electrical forces because only the divergence of polarization enters Maxwell’s first equation, relating charge and electric forces and field. The curl of any function can be added to a polarization field **P** without changing the electric field at all. The divergence of the curl is always zero. Additional information is needed to specify the curl and thus the structure of the **P** field. When the structure of charge changes substantially with the local electric field, the induced charge is a nonlinear and time dependent function of the field and **P** is not a useful framework to describe either the electrical or structural basis-induced charge. In the nonlinear, time dependent case, models must describe the charge distribution and how it varies as the field changes. One class of models has been used widely in biophysics to describe field dependent charge, i.e., the phenomenon of nonlinear time dependent induced charge, called ‘gating current’ in the biophysical literature. The operational definition of gating current has worked well in biophysics for fifty years, where it has been found to makes neurons respond sensitively to voltage. Theoretical estimates of polarization computed with this definition fit experimental data. I propose that the operational definition of gating current be used to define voltage and time dependent induced charge, although other definitions may be needed as well, for example if the induced charge is fundamentally current dependent. Gating currents involve substantial changes in structure and so need to be computed from a combination of electrodynamics and mechanics because everything charged interacts with everything charged as well as most things mechanical. It may be useful to separate the classical polarization field as a component of the total induced charge, as it is in biophysics. When nothing is known about polarization, it is necessary to use an approximate representation of polarization with a dielectric constant that is a single real positive number. This approximation allows important results in some cases, e.g., design of integrated circuits in silicon semiconductors, but can be seriously misleading in other cases, e.g., ionic solutions.

## 1. Introduction

When forces are applied to matter, the distribution of mass changes. Similarly, when electrical forces are applied matter with charge, the distribution of charge changes.

The electric field $E\left(x,y,z|t;\;\rho_{Q}\left(x,y,z|t;E\right)\right)$ changes the spatial distribution of charge P(x,y,z|t;E) producing polarization that has a central role in electrodynamics. In general, the change in charge distribution induced by the electric field will depend on time and electric field in a complex nonlinear way. We will discuss that situation later. But even when the induced charge is that of a polarization field characterized by a single dielectric constant (a real number), the actual definition of the polarization field P(x,y,z|t;E) is problematic, as major textbooks point out. Purcell and Morin [1], p. 500–507, show how the same structure can be described by different fields P(x,y,z|t). They conclude “The concept of polarization density P is more or less arbitrary” (slight paraphrase of [1], p. 507) and leads to an auxiliary variable that “is an artifice that is not, on the whole, very helpful” [1], p. 500.

Feynman shares this view. Feynman’s text says (on p. 10–17 of [2]) “One more point should be emphasized. An equation like $D=\varepsilon_{r}\varepsilon_{0}E$ is an attempt to describe a property of matter. But matter is extremely complicated, and the equation is in fact not correct.”, as he then explains in some detail [3]. (Zangwill [2] uses quantum electrodynamics (p. 160) to deal with P and avoids (p. 44) the auxiliary variable D**.** He concentrates on the fundamental variable E, as we do here.) Neither Purcell nor Feynman propose a general explanation for the ambiguity in P.

The significance of the Purcell and Morin and Feynman’s statements is great. If the concept of polarization is ‘more or less arbitrary’ (Purcell and Morin’s words); and the distinction between bound and free charge is ‘ambiguous’, then the formulation of the Maxwell equations in textbooks is ambiguous and arbitrary.

I hope it is not necessary to say the obvious: something as important as the Maxwell equations should not be presented in a way that two Nobel Laureates (Purcell and Feynman) think is ambiguous and arbitrary (their words, not mine). It seems that “…the conventional theory of electrodynamics inside matter needs to be redesigned”: p. 13 of [4].

A general explanation is presented here following Griffiths, Ch. 4, [5]. The ambiguity in the definition of polarization arises from a mathematical property of vector fields and not from a particular physics or structure of charges. Only the divergence of the polarization field enters into the equations for the electric field E and so very different functions can be added to P without changing the observable electric field. Specifically, the curl of any function can be added to **P** without changing the electric field because the divergence of the curl of any function is zero. Thus, measurements of E cannot determine the polarization field P uniquely. Different structures of polarization charge can give the same electric field and so measurements of the electric field cannot determine the structures producing polarization or there the structures of charge itself.

A paradigm widely used in biophysics to define gating current allows resolution of this ambiguity in many cases beyond biophysics. This paradigm cannot be universally applied but when it can be applied it is very useful. The dependence of polarization on the electric field is as complicated as the motions of matter in an electric field. These motions are nearly as complicated as the motions of matter in general. It is unlikely that any single paradigm will be universal. Nonetheless, the gating current paradigm of biophysics may be generally useful and will surely make specific what is needed for paradigms in general.

The paradigm of biophysics was developed to resolve the nonlinear displacement (i.e., capacitive) current of nerve that Hodgkin and Huxley [6] suggested might be the voltage sensor of nerve. This ‘gating current’ was measured in nerve [7] using a paradigm developed by Schneider and Chandler [8,9] and significantly improved by Bezanilla and Armstrong [10,11] and has been studied in great detail [7,12,13,14,15,16,17,18,19] because of the insight it gives [17,18,20,21] into the physical mechanism of conformation change in a most important biological protein and process. The conformation change of the voltage sensor determines many properties of the action potential, which is the signal used by the nervous system, skeletal and cardiac muscle to send signals more than a few micrometers.

The ambiguity of P arises from the history of electrodynamics, in my view. Faraday and Maxwell thought all charge depends on the electric field ([3], p. 36; [22,23,24]. All charge would then be polarization.

Maxwell used the D and P fields as fundamental dependent variables. Charge only appeared as polarization, usually over-approximated [25,26,27,28,29,30,31,32,33,34,35] by a dielectric constant $\varepsilon_{r}$ that is a single real positive number. Charge independent of the electric field was not included, because the electron had not been discovered: physicists at Cambridge University (UK) did not think that charge could be independent of the electric field. The electron was discovered some decades later, in Cambridge, ironically enough [36,37]. (Thomson’s monograph“intended as a sequel to Professor Clerk-Maxwell’s Treatise on electricity and magnetism” [38] does not mention charge, as far as I can tell. Clarendon Press: 1893. “intended as a sequel to Professor Clerk-Maxwell’s Treatise on electricity and magnetism”; does not mention charge, as far as I can tell. Faraday’s chemical law of electrolysis was not known and so the chemist’s ‘electron’ postulated by Richard Laming and defined by Stoney [39] was not accepted in Cambridge as permanent charge, independent of the electric field. It is surprising that the physical unit ‘the Faraday’ describes a quantity of charged particles unknown to Michael Faraday. Indeed, he did not anticipate the existence or importance of permanent charge on particles or elsewhere.) It then became apparent to all that the permanent charge of an electron is a fundamental source of the electric field. The electron and permanent charge must be included in the equations defining the electric field, e.g., Equations (1) and (6) as it is in every textbook I have examined.

For physicists today, the fundamental electrical variable is the E field that describes the electric force on an infinitesimal test charge. D and P fields are auxiliary derived fields that many textbooks think unnecessary, at best.

## 2. Theory

**Figure 1:**
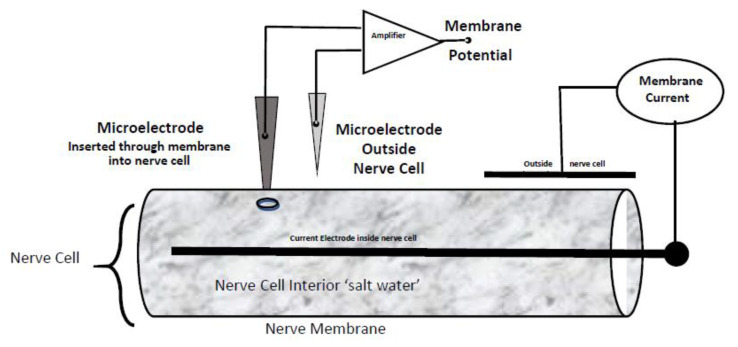
Setup for estimating the potential across the membrane of a cylindrical nerve cell while measuring the current through the membrane.

**Figure 2:**
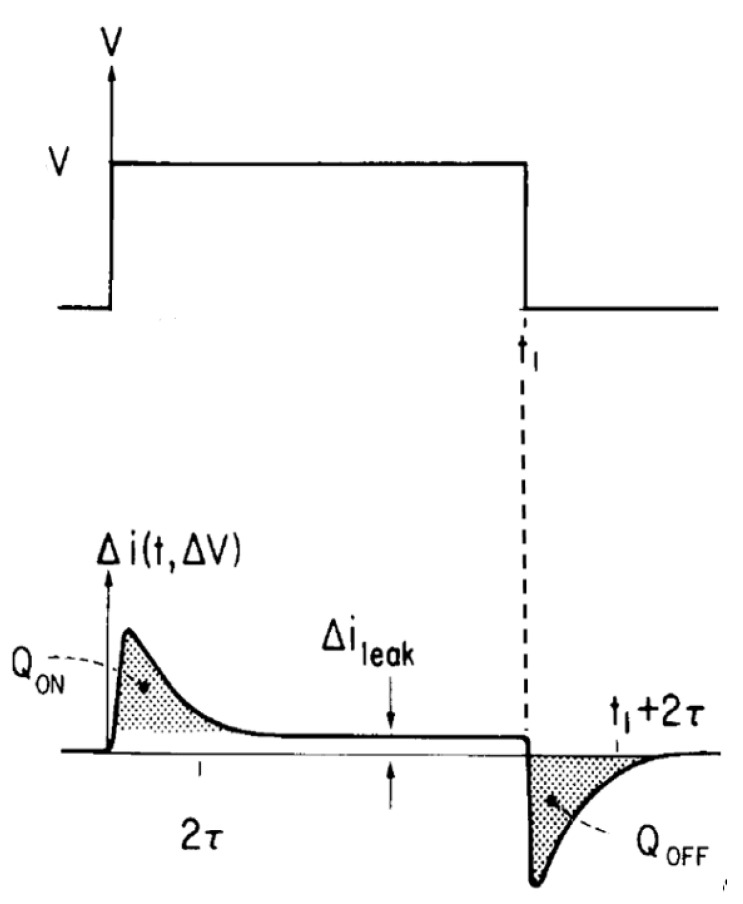
shows the response to a step function change in potential and the charges measured that are proposed as an operational definition of polarization.

The setup used here is described in many fine textbooks and so detail is omitted [1,2,3,4,5,40,41]. The specifics of the setup used to measure gating currents is described later, see Figure 1 and Figure 2.

Maxwell’s first equation for the composite variable D relates the ‘free charge’ $\rho_{f}\left(x,y,z|t\right)$, units cou/m^3^, to the sum of the electric field E and polarization P. It is usually written as

(1) $$div\;D\left(x,y,z|t\right)\;={\;\rho }_{f}\left(x,y,z|t\right)$$

(2) $$D\left(x,y,z|t;E\right)≜\;\varepsilon_{0}\;E\left(x,y,z|t\right)+P\left(x,y,z|t;E\right)$$

The physical variable E that describes the electric field is not visible in the classical formulation Equation (1). Maxwell embedded polarization in the very definition of the dependent variable $D≜\;\varepsilon_{0}\;E+P.$ $\varepsilon_{0}$ is the electrical constant, sometimes called the ‘permittivity of free space’. Polarization is described by a vector field P with units of dipole moment per volume, cou-m/m^3^, that can be misleadingly simplified to cou-m^−2^. The charge $\rho_{f}$ cannot depend on D or E in traditional formulations and so $\rho_{f}$ is a permanent charge.

When Maxwell’s first equation is written in a style appropriate since the discovery of the electron E is the dependent variable, as textbooks make clear. The source terms are $\rho_{f}$ and the divergence of P.

(3) $$\varepsilon_{0}div\;E\left(x,y,z|t\right)=\rho_{f}\left(x,y,z|t\right)-div\;P\left(x,y,z|t;E\right)$$

P does not have the units of charge and should not be called the ‘polarization charge’. P does not enter the equation by itself. Only the divergence of P appears on the right-hand side of Equation (3).

D(x,y,z|t) and the polarization P(x,y,z|t) are customarily over-approximated in classical presentations of Maxwell’s equations: the polarization is assumed to be proportional to the electric field, independent of time.

(4) $$P\left(x,y,z|t\right)≜\;\left(\varepsilon_{r}-1\right)\varepsilon_{0}\;E\left(x,y,z|t\right)$$

(5) $$D\left(x,y,z|t\right)≜\;\varepsilon_{r}\varepsilon_{0}E\left(x,y,z|t\right)$$

The proportionality constant $\left(\varepsilon_{r}-1\right)\varepsilon_{0}$ involves the dielectric constant $\varepsilon_{r}$ which must be a single real positive number if the classical form of the Maxwell equations is taken as an exact mathematical statement of a system of partial differential equations. If $\varepsilon_{r}$ is generalized to depend on time, or frequency, or the electric field, the form of the Maxwell equations changes. If $\varepsilon_{r}$ is generalized, traditional equations cannot be taken literally as a mathematical statement of a boundary value problem. They must be changed to accommodate the generalization.

Polarization and thus $\varepsilon_{r}$—however generalized—depend on time or frequency in complex ways in all matter as documented in innumerable experiments [33,34,35,42,43,44]. Many of the most interesting applications of electrodynamics arise from the dependence of polarization and $\varepsilon_{r}$ on field strength.

$\varepsilon_{r}$ should be taken as a constant only when experimental estimates, or theoretical models are not available, in my view.

It is difficult to imagine a physical system in which the electric field produces a change in charge distribution independent of time (see examples shown towards the end of Discussion). The time range in which Maxwell’s equations are used in the technology of our computers, smartphones, and video displays starts around $10^{-10}$ s. The time range in which Maxwell’s equations are used in biology start around $10^{-15}$ s in simulations of the atoms that control protein function. The time range of the X-rays that determine protein structure is $\sim 10^{-19}\;$s. The time range used to design and operate the synchrotrons that generate X-rays is very much faster than that, something like $10^{-23}\;$s. The Maxwell equations describe experiments to many significant figures over this entire range.

It is evident that a dielectric constant $\varepsilon_{r}$ independent of time is an inadequate over-approximation in many cases of practical interest today, in biology, engineering, chemistry, and physics.

Maxwell’s first equation for E is well described in many textbooks, although the inadequacies of the usual representation of polarization with a single dielectric constant are not emphasized, if mentioned at all. Students are then often unaware of the over-approximation, particularly if they have a stronger background in biology or mathematics than the physical sciences.

(6) $$\varepsilon_{r}\varepsilon_{0}div\;E\left(x,y,z|t\right)=\rho_{f}\left(x,y,z|t\right)$$

Polarization is particularly well described in Griffiths [5].

It is wise, in my view to combine the fields on the right-hand side of Equation (3) with the definition

(7) $$\rho_{Q}\left(x,y,z|t;E\right)\;≜\rho_{f}\left(x,y,z|t\right)-div\;P\left(x,y,z|t;E\right)$$

yielding the version of Maxwell’s first law that does not involve a polarization field P(x,y,z|t) at all.

(8) $$\varepsilon_{0}div\;E\left(x,y,z|t\right)=\rho_{Q}\left(x,y,z|t;E\right)$$

We adopt this version of Maxwell’s first equation here.

## 3. Results

The traditional formulation of the differential equations shown in Equations (1) and (6) is ambiguous in an important way (Integral forms of the Maxwell equations show more clearly the need for boundaries. They display the charge on the surface as an integral and explicit part of the general solution of Poisson’s equation for the electrical potential, for example). They do not mention the shape or boundaries of the regions in question. In fact, if P varies from region to region, but is constant within each region, charge is absent within each region: when P is constant, div P=0. Charge accumulates only at the boundaries of the regions. In many situations involving dielectrics, including most of those described in classical textbooks Only the boundary charge has effects on the Maxwell Equations (1) and (6). The P field in the Maxwell Equation (7), and implied in Equations (1) and (6), is zero; only the boundary values of P are important and they are not visible in the Maxwell Equations (1) and (6) themselves.

We turn now to applications in biology where the issue of charge at boundaries is particularly important, not to say that it is unimportant in semiconductor devices as well. Dielectric boundary charges have a particular role in biological systems involving membranes or proteins. The membrane capacitance, so important in determining the electrical properties of cells, particularly cells with action potentials like nerve and muscle, is a boundary phenomenon. Boundary charges are of great importance in channel proteins that allow (nearly catalyze) ion flow through membranes, see Appendix A on Proteins and [45].

Turning back to classical electrodynamics, we remember that most of the properties of dielectric rods studied by Faraday—and predecessors going back to Benjamin Franklin, if not earlier—arise from the dielectric boundary charges. Textbooks typically spend much effort teaching why polarization charge appears on dielectric boundaries in systems with constant P where div P=0 (e.g., Ch. 6 of [3]). Students wonder why regions of dielectrics without polarization charge have polarization charge on boundaries.

A general principle is at work here: a field equation in itself—like Equations (1) and (6) that are partial differential equations without boundary conditions—is altogether insufficient to specify an electric field. A model is needed that has boundary conditions. Applications of electrodynamics to biology, electrochemistry, and semiconductors are not useful until they specify models and boundary conditions that realistically describe the system of interest.

The model needs to include an explicit structure. It needs to describe the spatial variation of P. Indeed, the spatial variation of P may be a main determinant of properties [46,47,48] in (for example) many biological systems (e.g., channels), electrochemical systems (electrodes of batteries), and semiconductor devices. Without specifying boundary conditions (defined explicitly in specific structures), using P in the differential Equation (7), and implied in Equations (1) and (6), is ambiguous and confusing. Indeed, using P without boundary conditions is so incomplete that it might be called incorrect.

The general nature of the ambiguity in P becomes clear once one realizes that:

(9) $$\mathrm{Adding}\;curl\;\tilde{\mathbb{C}}\left(x,y,z|t\right)\mathrm{to}\;P\left(x,y,z|t\right)\;\mathrm{in}\;\mathrm{Maxwell}\text{’}s\;\mathrm{first}\;\mathrm{equation},\;\mathrm{Equation}\;\left(7\right)$$

changes nothing (Ch. 4 of [5]) because [49,50]

(10) $$div\;curl\;\tilde{\mathbb{C}}\left(x,y,z|t\right)\;\equiv 0\;;$$

The ambiguity in P in the Maxwell differential equations means that any model $P_{model}\left(x,y,z|t\right)$ of polarization can have $curl\;\tilde{\mathbb{C}}\left(x,y,z|t\right)$ added to it, without making any change in the div P(x,y,z|t) in Maxwell’s first equation (7), and implied in Equations (1) and (6).

In other words, the polarization div P(x,y,z|t) in Maxwell’s first Equations (7), and implied in Equations (1) and (6), does not provide a unique structural model of polarization $P_{model}\left(x,y,z|t\right)$. In particular, a model drawn from an atomic detail structure can be modified by adding a polarization $\tilde{\mathbb{P}}\left(x,y,z|t\right)≜curl\;\tilde{\mathbb{C}}\left(x,y,z|t\right)$ to its representation (i.e., ‘drawing’) of polarization without changing electrical properties at all: $div\;P\equiv div\;\left(P+\tilde{\mathbb{P}}\right)$.

Models of the polarization $P_{model}^{1}$ and $P_{model}^{2}$ of the same structure written by different authors may be strikingly different but they can give the same electrical results even though the models can appear to be very different. The $curl\;\tilde{\mathbb{C}}\left(x,y,z|t\right)$ field can be quite complex and hard to recognize in a model, particularly for structural biologists who may not be comfortable with vector calculus and its **curl** and **div** operators. The two models $P_{model}^{1}$ and $P_{model}^{2}$ produce the same charge distribution $div\;P_{model}^{1}$ and div $P_{model}^{2}$ in Maxwell’s first equation Equation (11) and so they cannot be distinguished by electrical measurements.

As we have seen, the **P** field is arbitrary, as certainly has been known previously Ch. 4 of [5]. Purcell and Morin [1], see pp. 500–507, describe structural models and ways to construct different fields P(x,y,z|t) from the same structure as stated in the introduction to this paper. **P** fields are not unique.

Purcell and Morin are not guilty of overstatement—indeed they may be guilty of understatement—when they say “The concept of polarization density P is more or less arbitrary” (slight paraphrase of [1], p. 507) and the D field is “is an artifice that is not, on the whole, very helpful” [1], p. 500.

The classical approach criticized by Purcell and Morin [1] does not allow unique specification of a polarization field P(x,y,z|t) from electrical measurements.

An arbitrary artificial formulation is prone to artifact and likely to produce misunderstanding and unproductive argument: “what is the true description of a dielectric object (e.g., protein)?” is a question likely to arise and be unanswerable if the polarization field **P** is itself not unique.

The P(x,y,z|t) of classical theory is not a firm foundation on which to build an understanding of the structural basis of the phenomena of polarization, or the electrodynamics of matter, with problems particularly apparent in the understanding of the polarization arising from the structure of proteins (see Appendix A).

It seems clear that most formulations of electrodynamics of dielectrics in classical textbooks are “more or less arbitrary” and depend on an “artifice” (quotations from Feynman and Purcell and Morin). Because dielectrics, polarization and a dielectric constant (as a single real number) are central to the classical treatments of electrodynamics, the conclusion (p. 13) of a modern monograph on electrodynamics, using mathematics (exterior differential forms) appropriate for relativistic theories of electrodynamics, [4] quoted previously seems worth restating “We believe that the conventional theory of electrodynamics inside matter needs to be redesigned”. That redesign begins with a revised treatment of polarization that reflects the ambiguity of the curl, see [5]. Ambiguity and its problems can be avoided if Maxwell’s First Equation is rewritten without a polarization field P(x,y,z|t) as shown previously in Equation (8). The phenomena of polarization—the response of charges to an electric field—is then included in a variable $\rho_{Q}\left(x,y,z|t;E\right)$, specifically as (part of) its dependence on E:

(11) $$div\;\varepsilon_{0}E\left(x,y,z|t\right)\;=\rho_{Q}\left(x,y,z|t;E\right)\;$$

Here $\rho_{Q}\left(x,y,z|t;E\right)$ describes all charge whatsoever, no matter how fast, small or transient are their movements, including what is usually called dielectric charge and permanent charge, as well as charges driven by other fields, like convection, diffusion or temperature. The charge $\rho_{Q}$ can be parsed into components in many ways (see Equations (1), (3), (6) and (8) and [43,51]). Updated formulations of the Maxwell differential equations [43,51] are needed, in my opinion, to avoid the problems produced by ambiguous P and over-simplified $\varepsilon_{r}$.

We turn now to a quite different property of charge matter, the flow of charges.

Most applications of electrodynamics involve flow. The most prominent application of electrodynamics is surely computational and semiconductor electronics [52,53,54,55,56,57,58,59,60,61] and that involves flow, usually described by Kirchhoff’s current law. Semiconductor electronics has remade our world increasing computer power by nearly 10^9^× in the last seventy years [62,63,64,65,66,67]. Biology and electrochemistry (batteries) scarcely exist without flow: what physical chemists call equilibrium (no flows of any kind) is hardly worth studying in biological or electrochemical systems. Unlike thermodynamics, electrodynamics nearly always involves flow.

Thus, we study the flux of charges $\rho_{Q}$ as well as their density. Maxwell’s second equation describes the flow of charges, electrical current, and the magnetic field. It is understandable that Maxwell—and his Cambridge contemporaries and followers—had difficulty understanding current flow when their models did not include permanent charge, electrons or their motions.

Maxwell’s extension of Ampere’s law describes the special properties of current flow $J_{total}$ (Equation (13) that make it so different from the flux of matter. Maxwell’s field equations include the ethereal current $\varepsilon_{0}\partial E/\partial t$ that makes the equations resemble those of a perfectly incompressible fluid: the ethereal current always exists, whether matter is present or not, unlike the dielectric current $\left(\varepsilon_{r}-1\right)\varepsilon_{0}\partial E/\partial t$ that exists only when matter is present.

Maxwell’s field equations describe the incompressible flow $J_{total}$ over the dynamic range of something like $10^{16}$ that is safely accessible within laboratories. The dynamic range of the Maxwell equations is much larger if one includes the interior of stars, and the core of galaxies in which light is known to follow the same equations of electrodynamics as in our laboratories.

Maxwell’s field equations are different from material field equations (like the Navier–Stokes equations) because they are meaningful and valid universally [68], both in a vacuum devoid of mass and matter and within and between the atoms of matter [43].

The ethereal current $\varepsilon_{0}\partial E/\partial t$ responsible for the special properties of Maxwell’s equations arises from the Lorentz (un)transformation of charge. Charge does not vary with velocity, unlike mass (this is the mass that determines inertia, called the ‘relativistic mass’ nowadays. This was the meaning of the word ‘mass’ in Einstein’s original papers, presumably because he wanted an operational definition of ‘mass’ that was based on the observable properties, inertia and momentum, and that was independent of Lorentz transformations, and theoretical considerations) [69], length, and time, all of which change dramatically as velocities approach the speed of light, strange as that seems. This topic is explained in any textbook of electrodynamics that includes special relativity. Feynman’s discussion of ‘The Relativity of Electric and Magnetic Fields’ was an unforgettable revelation to me as a student, see Section 13-6 of reference [2]: an obervers moving at the same speed as a stream of electrons sees zero current, but the forces measured by that observer are the same as the forces measured by an observed who is not moving at all. The moving observer describes the force as an electric field E(x,y,z|t). The unmoving observer describes the force as a magnetic field B(x,y,z|t). The observable forces are the same, whatever they are called, according to the principle and theory of relativity. (The principle and theory of relativity are confirmed to many significant figures every day in the GPS (global positioning systems) software of the map apps on our smartphones, and in the advanced photon sources (synchrotrons) that produce X-rays to determine the structure of proteins).

The ethereal current reveals itself in magnetic forces which have no counterpart in material fields. The ethereal current is apparent in the daylight from the sun, that fuels life on earth, and in the night light from stars that fuels our dreams as it decorates the sky. The ethereal current is the term in the Maxwell equations that produces propagating waves in a perfect vacuum like space.

Magnetism B is described by Maxwell’s version of Ampere Law, Maxwell’s Second Equation:

(12) $$\frac{1}{\mu_{0}}\;curl\;B=\;J_{Q}+\;\varepsilon_{0}\frac{\partial E}{\partial t}$$

(13) $$J_{total}≜\;J_{Q}+\;\varepsilon_{0}\frac{\partial E}{\partial t}$$

(14) $$\frac{1}{\mu_{0}}\;curl\;B=J_{total}$$

If we are interested in flux and current, we must turn to Maxwell’s second equation and deal explicitly with magnetism, even if magnetic fields themselves do not carry significant energy (as in almost all biological applications). Only by dealing with Maxwell’s second equation can we derive conservation of total current and compare it with the conservation of charge. Indeed, the derivation of the continuity equation used here depends on equations involving the magnetic field.

Note that $J_{Q\;}$ includes the movement of all charge $\rho_{Q}$ with mass, no matter how small, rapid or transient. It includes the movements of charge classically approximated as the properties of an ideal dielectric. It describes all movements of the charge described by $\rho_{Q}\left(x,y,z|t;E\right)$; $\rho_{f}$ is one of the components of $\rho_{Q}.$ Indeed, $J_{Q}$ can be written in terms of $v_{Q}$ the velocity of mass with charge. In simple cases, such as a plasma of ions each with charge $Q_{Q}$

(15) $$J_{Q\;}=v_{Q}Q_{Q}N_{Q}$$

where $Q_{Q}$ is the charge per particle and $N_{Q}$ is the number density of particles. In a mixture, sets of fluxes $J_{Q}^{i}$, velocities $v_{Q}^{i}$, charges $Q_{Q,}^{i}$ number densities $N_{Q}^{i}$, and charge densities $\rho_{Q\;}^{\;i}$ are needed to keep track of each elemental species i of particles. Plasmas are always mixtures because they must contain both positive and negative particles to keep electrical forces within safe bounds, as determined by (approximate) global electroneutrality.

In cases other than plasmas, the relationship of $J_{Q\;},\;J_{total}$ and $Q_{Q}$ to material properties is complex. The relationship often involves convection and diffusion fields and extends over a range of scales from atomic to macroscopic, in both space and time. For example, the Maxwell equations do not describe charge and current driven by other fields, like convection, diffusion, or temperature. They do not describe constraints imposed by boundary conditions and mechanical structures. Those must be specified separately. If the other fields, structures, or boundary conditions involve matter with charge, they will respond to changes in the electric field. The other fields and constraints thus contribute to the phenomena of polarization and must be included in a description of it, as we shall discuss further below in the examples shown towards the end of Discussion. The theory of complex fluids has dealt with many such cases, often with the label ‘micro macro’, spanning scales, connecting micro (even atomic) structures with macro phenomena.

The charge density $\rho_{Q}$ and current $J_{total}$ can be parsed into components in many ways, some helpful in one historical context, some in another. References [33,43,51,70,71,72,73,74,75] define and explore those representations in tedious detail. Simplifying those representations led to the treatment in this paper.

Maxwell’s Ampere’s law Equation (12) implies two equations of great importance and generality. First, it implies a continuity equation that describes the conservation of charge with mass. The continuity equation is the relation between the flux of charge with mass and density of charge with mass.

**Derivation**: Take the divergence of both sides of Equation (12), use div curl = 0 [49,50], and get

(16) $$div\;J_{Q}=div\;\left(-\;\varepsilon_{0}\frac{\partial E}{\partial t}\right)=\;-\;\varepsilon_{0}\frac{\partial }{\partial t}div\;E$$

when we interchange time and spatial differentiation.

However, we have a relation between div E and charge $\rho_{Q}$ from Maxwell’s first equation, Equation (11), giving the Maxwell Continuity Equation:

(17) $$div\;J_{Q}\;=-\;\varepsilon_{0}\;\varepsilon_{0}\frac{\partial \rho_{Q}}{\partial t}$$

(18) $$div\;\left(v_{Q}Q_{Q}N_{Q}\right)\;=-\;\varepsilon_{0}\frac{\partial \rho_{Q}}{\partial t},$$

for a biophysical or astrophysical plasma of ions.

Note that sets of fluxes $J_{Q}^{i}$ and sets of charge densities $\rho_{Q\;}^{\;i}$ are needed to keep track of each elemental species i of particles in a mixture, along with sets of velocities $v_{Q}^{i}$, charges $Q_{Q,}^{i}$ and number densities $N_{Q}^{i}$, as described near Equation (15).

Maxwell’s Ampere’s law Equation (12) implies a second equation of great importance. Indeed, it is this equation that allows the design of the one-dimensional branched circuits of our digital technology using the relatively simple mathematics of Kirchhoff’s current law [72,74].

**Derivation**: Taking the divergence of both sides of Maxwell’s Second law Equation (12) yields Conservation of Total Current

(19) $$div\;J_{total}≜div\;\left(J_{Q}\;+\varepsilon_{0}\frac{\partial E}{\partial t}\;\right)=0$$

(20) $$div\;J_{total}=0$$

or

(21) $$div\;J_{total}≜div\;\left(v_{Q}Q_{Q}N_{Q}J_{Q}\;+\varepsilon_{0}\frac{\partial E}{\partial t}\;\right)=0$$

It is easy to overlook the importance of one-dimensional systems. They may seem trivial, almost unworthy of analysis using the powerful beauty of vector calculus. However, one-dimensional systems are of great importance despite, or because of their simplicity.

Nearly all of our electronic technology occurs in one-dimensional systems, networks of branching one-dimensional conductors. Our electronic technology is driven by batteries that are one-dimensional systems. Our technology is at the hands of animals, humans in which all information transfer is done by one-dimensional circuits, unbranched in ion channels, and barely branched in nerve cells. Branched one-dimensional systems describe the metabolic pathways of biological cells that make life possible.

The importance of one-dimensional systems may come from their design. The design of one-dimensional systems is relatively easy for engineers or evolution. Design requires Kirchhoff’s laws and little else. One-dimensional systems are widely used for another reason. They are reliable. The dimensionality of these circuits rules out spatial singularities. Systems are more robust when steep slopes near infinities are not present to create severe sensitivity.

Kirchhoff’s laws are used to design semiconductor circuits that work over an enormous range of sizes and times, from say $10^{-10}\;s\;\mathrm{to}\;\mathrm{many}\;\mathrm{minutes},\;\mathrm{from}\;10^{-19}\;m\;\mathrm{to}\;10^{4}\;m\;\mathrm{or}\;\mathrm{longer}.$ Current flow over these ranges of time space involves a wide range of physics, described by many constitutive equations.

Current is not just the movement of point permanent charges as assumed in the textbook derivations of Kirchhoff’s current law I have consulted, both in electrical engineering and electrodynamics. The derivations of Kirchhoff’s current law are usually restricted to the simplest case of the long-time translation of point permanent charges, although it is very well known that is a poor model for current flow under conditions actually found in the integrated circuits of our digital technology. It is possible to show, however, that current flow in one-dimensional systems can be described accurately by a simple generalization of Kirchhoff’s current law that arises naturally from the treatment of Maxwell’s equations found in this paper: all the $J_{total}$ that flows into a node must flow out [51,72,73,74]. This result seems to be rather new, although of course it seems elementary and obvious. Indeed, it is so obvious that it must exist somewhere in the literature, even though I do not know where.

Kirchhoff’s current law take on simplest form in unbranched one-dimensional systems. Unbranched one-dimensional systems are important despite their utter simplicity. Indeed, the ion channels of biological systems control a wide range of biological function and are unbranched one-dimensional series systems. They cannot be considered degenerate. Nor can be the diodes of electronic technology that are also series systems. However, the greatest importance of unbranched one-dimensional systems may be the insight they give to the importance of the ethereal current $\varepsilon_{0}\partial E/\partial t,\;\mathrm{as}\;\mathrm{we}\;\mathrm{shall}\;\mathrm{soon}\;\mathrm{see}.$

Unbranched one-dimensional systems have components in series, each with its own current voltage relation arising from its microphysics. In a series one-dimensional system, the total current $J_{total}$ is equal everywhere at any time in every location no matter what the microphysics of the flux $J_{Q}$ of charge with mass. The current through a battery is an exceedingly complicated mixture of the microphysics of electrodes, ion movement and electron flow. If that battery is connected by a wire to a vacuum capacitor, the microphysics of the vacuum capacitor $i_{capacitor}\;=\;A_{rea}\varepsilon_{0}\partial E/\partial t,$ is as simple as the microphysics of the battery is complex, yet the total currents in the capacitor and the battery are equal at any time, in any conditions. Indeed, the microphysics of the wire linking the capacitor and the battery is totally different from the microphysics of the capacitor and battery. The microphysics of the wire actually resemble that of a waveguide at frequencies important in our digital integrated circuits. The microphysics of the wire, capacitor and battery do not change the fact that the total current through each is exactly the same, always, at every location and at every time.

How can that possibly be true? The answer is found in the Maxwell equations. They can be solved for the electric field and magnetic fields that make the total currents equal.

The solutions of Maxwell’s equations ensure that the ethereal current $\varepsilon_{0}\partial E/\partial t,\;$ and the other dependent variables, take on the values at every location and every time needed to make the total currents $J_{total}$ equal everywhere. A practical example, not difficult to build in any laboratory, including resistor, capacitor, diode, capacitor, cylinder of salt water, and wire is described in detail near Figure 2 of [73].

There is no spatial dependence of total current in a series one-dimensional system. No spatial variable or derivative is needed to describe total current in such a system [75], although of course spatial variables are needed to describe other variables, including (1) the density of mass with charge $Q_{Q}^{i}$ (2) the flux $J_{Q}$ of charge with mass (3) the electrical current $J_{total}^{i}$ of individual elemental species (4) the velocities, charge, and number densities $v_{Q},{\;Q}_{Q},{\;\rho }_{Q},\;$and $N_{Q}$.

It is important to realize that the flux of charge with mass $J_{Q}\;$ is not conserved, only the total current $J_{total}$ is conserved. Charges carry $J_{Q}$ can accumulate. In fact, $div\;J_{Q}=div\;\left(v_{Q}Q_{Q}N_{Q}\right)$ supplies the flow of charge that is the current $\partial \rho_{Q}\;/\partial t$ necessary to change $div\;\left(\varepsilon_{0}\partial E/\partial t\right)$ as described by the following continuity equation.

(22) $$div\;J_{Q}=div\;\varepsilon_{0}\frac{\partial E}{\partial t}=\frac{\partial \;}{\partial t}div\;\left(\varepsilon_{0}E\right)=\frac{\partial \rho_{Q}}{\partial t}.$$

That is to say, $J_{Q}$ can accumulate as $Q_{Q}.$ Total current $J_{total}$ cannot accumulate, not at all, not anywhere, not at any time.

Because conservation of total current applies on every time and space scale, including those of thermal motion, the properties of $J_{Q}$ differ a great deal from the properties of $J_{total}$. For example, in one-dimensional channels, the material flux $\;J_{Q}$ can exhibit all the complexities of a function of infinite variation, like a trajectory of a Brownian stochastic process, that reverses direction an uncountably infinite number of times in any interval. A Brownian trajectory of a Brownian stochastic process is a continuous function that does not have a (well defined) time derivative anywhere.

In marked contrast to the infinite variation of $J_{Q}$, the electrical current $J_{total}$ has no spatial variation at all. It is spatially uniform [75].

The fluctuations of $\varepsilon_{0}\partial E/\partial t$ (in time and space) and other variables are exactly what are needed to completely smooth the infinite fluctuations of $J_{Q}$ into the spatially uniform $J_{total}$.

Maxwell’s equations serve as the perfect low pass (spatial) filter converting the infinite variation of Brownian motion into a spatial constant, as strange as that seems.

These universal and exact properties of Maxwell’s equations are hidden in the usual treatment of Maxwell’s equations. The usual treatment includes a grossly approximate treatment of polarization as the property of a perfect dielectric. Everyone knows how bad this approximation is, so everyone understands that Maxwell’s equations as usually written are not universal or exact. They are as sloppy as is the dielectric constant as a description of the polarization of matter.

***ONLY*** when Maxwell’s equations are written without a dielectric constant, with a perfectly general treatment of induced charge, does it become clear that Maxwell’s equations are universal and exact independent of any property of matter.

How then is polarization included in a modified version of the Maxwell equations that does not include a dielectric constant. One needs an explicit model of polarization appropriate for the system of interest.

It is obvious that one cannot describe material flow unless one knows how matter moves in response to forces. It should be obvious that one cannot describe the flux of charges unless one knows how material charge moves in response to forces.

The use of a single real dielectric constant in Maxwell’s equations is no more necessary than the use of a single spring constant (i.e., elasticity) is in material equations. But Maxwell’s equations describe the total electrical current—that includes the ethereal current—not the flux of charges. Because of the ethereal current, Maxwell’s equations describe light in the vacuum of space between stars.

Because of the ethereal current, Maxwell’s equations are universal and exact. They describe total current as exactly as they describe anything, and their description of total current flow is entirely independent of the properties of matter. Total current flow depends on no constitutive equations, except perhaps the constitutive equation of a vacuum, more or less determined by special relativity. Electrodynamics are very different in this respect from the equations of material movement. They always depend on constitutive equations in important respects. The fundamental properties of electrodynamics do not depend on constitutive equations.

## 4. Discussion: From Electrodynamics to Biophysics and Back

A fundamental question arises with the updated version of Maxwell’s equations. How is the phenomenon of polarization included in Equation (11) and Equation (14)?

To answer this question, we first need a general paradigm to define polarization, even when dielectrics are far from ideal, when they might be time and frequency dependent, and voltage dependent as well. We need a paradigm that describes how the charge distribution varies with the electric field in as general a system as possible, including systems with charge movement driven by forces not in the Maxwell equations at all, such as convection and diffusion.

It seems obvious that a general paradigm cannot be found. After all the motions of matter in response to a change in electric field are more or less as complex as the motions of matter itself! Nonetheless, a paradigm of that may be helpful in many cases has been in use for many years, even if it is not perfectly general.

This problem has been addressed in membrane biophysics. A community of scholars has studied the nonlinear currents that control the opening of voltage sensitive protein channels for nearly fifty years, [7,12,13,14,15,16,17,18,19] inspired by [6]. They have developed protocols that may be useful in other systems, as they have been in biophysics. Schneider and Chandler followed by Bezanilla and Armstrong are responsible for this paradigm, more than anyone else [7,8,9].

The basic setup used in these experiments is that of an electrochemical cell modified to deal with a cylindrical cell as shown in Figure 1. Membrane potential is measured across a biological membrane, with defined concentrations on both sides of the membrane. Current is applied through electrodes to control the potential, in the classical voltage clamp set up of Cole [76] and Hodgkin, Huxley, and Katz [77,78]. It is best to apply that current in electrodes different from those that record membrane potential using a so-called four electrode setup [79,80,81], like those described in textbooks of electrochemistry.

I propose using the operational definition of ‘gating current’ used to define nonlinear, time and voltage dependent polarization by biophysicists as a useful setup and definition of many types of polarization. Obviously, this definition is not general, but the hope is that it may be generally useful.

The basic idea is to apply a set of step functions of potential across the system—in biology across the membrane—and observe the currents that flow. The currents observed are transients that decline to a steady value, often to near zero after a reasonable (biologically relevant) time. The measured currents are perfectly reproducible. If a pulse is applied, the charge moved (the integral of the current) can be measured when the voltage step is applied. The integration goes on until $t_{1}$ when the current $i_{leak}$ is nearly independent of time, often nearly zero. That integral is called the ON charge $Q_{ON}$.

When the voltage is returned to its initial value (the value that was present before the ON pulse), another current is observed that often has quite different time course [7,8,9], much more so than in Figure 1. The integral of that current is the OFF charge $Q_{OFF}$.

If $Q_{ON}=Q_{OFF}$, and the physical processes involved depend fundamentally on potential and not its time derivative, the biophysical paradigm is likely to be useful. In other cases, another paradigm is needed. If the current produced by the step in potential is in fact actually transient, the steady current will be what it was before the voltage step was applied. The transient will disappear with time as the word ‘transient’ implies. In that case it seems that the biophysical paradigm is not only useful but may even provide a unique definition of gating current and the corresponding polarization.

Gating current as measured in biophysical experiments depends on the membrane voltage before the step, as well as the voltage just after and during the step. It also depends separately on the voltage after the step, although Figure 1 does not illustrate the dependence documented in the literature [7,8,9]. The voltage and time dependence arises from the molecular motions underlying the gating current. The voltage and time dependence defines the mean molecular motions [7,16,17,19,21,82,83,84,85,86] and is called ‘the gating current’ in the biophysics literature.

If the ON charge is found experimentally to equal the OFF charge, for a variety of pulse sizes and range of experimental conditions, the current is said to arise in a nonlinear (i.e., voltage dependent) polarization capacitance and is interpreted as the movement of charged groups in the electric field. The charged groups move to one location after the ON pulse, and return to their original location following the OFF pulse. The charge is called ‘gating charge’, and the current that carries the charge is called ‘gating current’.

The macroscale current observed in the set-up is equal to the sum of the micro (actually atomic scale) currents carried by the charged groups inside a channel protein, even though the recording electrodes are remote from the protein. Indeed, there might be $10^{18}$ charged atoms (ions) between the electrodes and the protein.

The currents in the electrodes and the channel protein are equal because the setup is designed to be an unbranched one-dimensional circuit with everything in series. In a one-dimensional series setup the total current is equal everywhere in the series system at any one time, even though the total current varies significantly with time. The Maxwell equations guarantee spatial uniformity of total current (including the ethereal current $\varepsilon_{0}\partial E/\partial t$) independent of the microphysics of movement of charge (with mass): Figure 2 of [73], and [43,75,87]. The equality of current can be checked by measuring current in different locations in the experiment. The spatial equality of current needs also to be checked in simulations as in [18,21,88] because tiny inadvertent errors in numerical procedures or coding can produce substantial deviations from spatial equality and thus misleading artifacts. Imposing periodic boundary conditions on nonperiodic systems is another possible source of such artifacts.

If the currents reach a steady value independent of time, but not equal to zero, as in Figure 1, the signal is not transient, in the strict meaning of the word. In biophysics, the steady current $i_{leak}$ is then usually considered to flow in a resistive path that is time independent, but perhaps voltage dependent, in parallel with the path or device in which the gating charges $Q_{\mathrm{ON}}$ and $Q_{\mathrm{OFF}}$ flow. If the current does not reach a steady value, or if the areas in Figure 2 are not equal, the currents are not considered ‘capacitive’ and are interpreted as those through a time and voltage dependent ‘resistor’. This is a biological and biophysical assumption. It is not a physical or mathematical necessity. Thus, it is important to investigate the properties of the currents through the resistive path—e.g., those that are not transient and do not return to zero and those that make $Q_{\mathrm{ON}}\neq Q_{\mathrm{OFF}}$ by ***independent*** methods to see if they are time independent. In biophysics, currents can be done by blocking the resistive path with drugs, or with mutations of the channel protein. If the resistive currents are not time independent, the definition of $Q_{\mathrm{ON}}$ and $Q_{\mathrm{OFF}}$ in Figure 1 needs to be changed. Indeed, experiments of another type must be designed that allow separation of polarization from conduction currents. The simplest version of the biophysics paradigm then needs to be extended.

Clearly, this approach will only work if step functions can reveal all the properties of the underlying mechanism. If the underlying mechanisms depend on the time rate of change of voltage, step functions are clearly insufficient because ∂V/∂t, is zero or infinity but nothing else in a step function. In the classical language of membrane biophysics, the ionic conductances $g_{Na}$ and $g_{K}$ must not depend on the rate of change of voltage.

Much work has been conducted showing that step functions are enough to understand the voltage dependent mechanisms in the classical action potential of the squid axon [89,90,91], starting with [78], Figure 10 and Equation (11). Hodgkin kindly explained the significance of this issue to colleagues, including the author (around 1970). He explained the possible incompleteness of step function measurements: if sodium conductance had a significant dependence on ∂V/∂t, the action potential computed from voltage clamp data would differ from experimental measurements. He mentioned that this possibility was an important motivation for Huxley’s heroic hand integration [6] of the Hodgkin Huxley differential equations. Huxley confirmed this in a separate personal communication, Huxley to Eisenberg. Those computations and many papers since [89,90,91] have shown that voltage clamp data (in response to steps) is enough to predict the shape and propagation of the action potential in nerve and skeletal muscle. It should be clearly understood that such a result is not available for biological systems in which the influx of Ca^++^ drives the action potential and its propagation [92].

The conductance of the voltage activated calcium channel has complex dependence on the current through the channel because the concentration of Ca^++^ in the cytoplasm is so low (~10^−8^ M at rest) that the current almost always changes the local concentration in and near the channel on the cytoplasmic side. Those concentration changes, in turn, alter the gating and selectivity characteristics of the channel protein, as calcium ions are prone to do int many physical and biological systems, particularly at interfaces.

It seems unlikely that the resulting properties of voltage dependent calcium channels can be comfortably described by the same formalism [6] used for voltage-controlled sodium and potassium channels of nerve and skeletal muscle. That formalism uses variables that depend on membrane potential and not membrane current because Cole [93] and Hodgkin [94,95,96] guessed that neuronal action potentials were essentially voltage dependent, not current dependent. They found action potentials in ‘space clamped’ axons with wires down their middle [76,77,97,98] that ensured spatial uniformity of potential. These axons had very different patterns of current flow from normal axons, and so Cole and Hodgkin were confirmed in their view that the membrane processes generating the action potential were voltage dependent, much more than current dependent (personal communication Cole to Eisenberg 1960; Hodgkin to Eisenberg 1961, et al.).

Hodgkin, Huxley, Katz, and Cole did not know of action potentials driven by calcium channels [99,100,101,102,103], nor of the extraordinarily small concentration of calcium ions inside cells. There may of course be other reasons the formalism [6] is inadequate. In summary, experiments, theory, computations and perhaps simulations are needed to show that responses to steps of voltage allow computation of a calcium driven action potential.

The polarization protocol described here can be applied to simulations of polarization as well as experimental measurements of polarization. Indeed, the operational definition of polarization has been applied even when theories [18] or simulations are enormously complicated by atomic detail that includes the individual motions of thousands of atoms [21,88].

Another question of general interest is how does the polarization defined this way correspond to the polarization $-P=\left(\varepsilon_{r}-1\right)\varepsilon_{0}E$ in the classical formulation of the Maxwell Equations (7) and implied in Equations (1) and (6)? Does the estimated polarization equal P?

The answer is not pleasing. Polarization cannot be defined in general. The variety of possible responses of matter to a step of potential prevents a general answer. Indeed, a main point of this paper is that polarization must be defined by a protocol in a specific setting that specifies how the local electric field changes the distribution of charge.

Polarization cannot be defined in general because there are too many possible motions of mass with charge in response to a change in the electric field. Every possible motion of mass (with charge), including rotations and translations and changes of shape and density of charge, would produce a polarization. Polarization currents can be as complicated as the motions of matter.

In mechanical systems in general these issues do not attract much attention. It seems obvious that one must have a model and theory of how a system changes shape (and distribution of mass) when forces are applied. Seeking a general treatment is silly. In electrodynamics, for illogical reasons of history, tradition, and respect for our elders, scientists have sought the general treatment that would be considered silly for mechanical systems.

Scientists, certainly including me, have used the simple electromechanical model of an ideal dielectric to describe how charge moves in response to an electric field, using the name polarization to describe the phenomena. They have tried to apply it everywhere, as is seen because that model is embedded in the traditional formulation of the Maxwell equations found universally in textbooks.

It seems to me time to abandon this forlorn hope of a general description of the response of charged matter to a change in the electric field, and to move to a more reasonable approach, in which explicit models of the response of charge to the electric field are constructed, with different models for different systems.

Insight can be developed into various kinds of polarization by constructing ‘toy’ models of simple systems. Those models must specify the mechanical variables $v_{Q,}\;Q_{Q},\;\rho_{Q}$ and $N_{Q}$ (or their equivalent) and solve the field equations of mechanics, perhaps including diffusion, along with the Maxwell equations. The models are then studied using the operational definition of polarization, described previously (Figure 1) or other operational definitions more suitable for other systems. One can hope some of the models resemble some of the more elaborate models of polarization already in the literature [26,27,28,29,31,32,34].

Toy models might include:

(1) Simple electro-mechanical models, like a charged mass on a spring with damping.
(2) Ideal gases of permanently charged particles, i.e., biological and physical plasmas.
(3) Ideal gases of dipoles (point [104] and macroscopic), quadrupoles, and mixtures of dipoles and quadrupoles, that rotate and translate while some are attached by bonds that vibrate (see (1)). These mixtures should provide decent representations of liquid water in ionic solutions, if they include a background dielectric, even if the dielectric is over-approximated with a single dielectric constant $\varepsilon_{r}\left(H_{2}O\right)≅80$. Indeed, there is a substantial literature of such models, including [105,106] but one must be sure that the models include the unavoidable interactions of atoms, molecules, and structures often dominated by their electrodynamics. Atoms, molecules and structures are almost always charged and so never move independently. Their motions are correlated by the electric field, and those correlations are likely to dominate the properties of greatest interest in applications. Of course, the extensive analysis of these authors can be of great use once it is focused on issues and applications of interest and combined with experimental measurements (see (5) and (6) below).
(4) Molecular models of ionic solutions that include water as a molecule. It is best to use models that are successful in predicting the activity of solutions of diverse composition and content and include water and ions as molecules of unequal nonzero size [107].
(5) Classical models of impedance, dielectric, and molecular spectroscopy [26,27,28,29,31,32,34].
(6) Well-studied systems of complex fluids, spanning scales, connecting micro (even atomic) structures with macroscopic functions, often called ‘micro-macro models’ in the literature.

These examples, taken together, will help form a handbook of practical examples closely related to the classical approximations of dielectrics.

These problems have time dependent solutions except in degenerate, uninteresting cases. Time dependence poses particular problems for the classical formulations of Maxwell equations. As stated in [51] on p. 13.

“It is necessary also to reiterate that $\varepsilon_{r}$ is a single, real positive constant in Maxwell’s equations as he wrote them and as they have been stated in many textbooks since then, following [108,109,110]. If one wishes to generalize $\varepsilon_{r}$ so that it more realistically describes the properties of matter, one must actually change the differential Equation (6) and the set of Maxwell’s equations as a whole. If, to cite a common (but not universal) example, $\varepsilon_{r}$ is to be generalized to a time dependent function, (because polarization current in this case is a time dependent solution of a linear, often constant coefficient, differential equation that depends only on the local electric field), the mathematical structure of Maxwell’s equations changes”.

Perhaps it is tempting to take a short cut by simply converting $\varepsilon_{r}$ into a function of time $\varepsilon_{r}\left(t\right)$ in Maxwell’s equations, as classically written. “Solving the equations with a constant $\varepsilon_{r}$ and then letting $\varepsilon_{r}$ become a function of time creates a mathematical chimera that is not correct. The chimera is not a solution of the equations.” The full functional form, or differential equation for $\varepsilon_{r}\left(t\right)$ must be written and solved together with the Maxwell equations. This is a formidable task in any case, but becomes an even more formidable challenge if convection or electrodiffusion modify polarization, as well as the electric field.

If one confines oneself to sinusoidal systems (as in classical impedance or dielectric spectroscopy [27,42,111,112]), one should explicitly introduce the sinusoids into the equations and not just assume that the simplified treatment of sinusoids in elementary circuit theory [113,114,115,116,117] is correct. It is not at all clear that Maxwell’s equations joined with constitutive equations; and boundary conditions always have steady state solutions in the sinusoidal case. The Maxwell equations joined with diffusion and convection equations (like Navier–Stokes [118,119,120,121,122,123,124,125,126,127,128,129,130,131,132,133,134,135] or PNP = Poisson Nernst Planck = drift diffusion [52,53,55,57,59,61,123,136,137,138,139,140,141,142,143,144,145]) certainly do not always have solutions that are linear functions of just the electric field [146,147,148,149].”

It seems clear that the classical Maxwell equations with the over-approximated dielectric coefficient $\varepsilon_{r}$ cannot emerge in the time dependent case. Of course, the classical Maxwell equations cannot emerge when polarization has a nonlinear dependence on the electric field, or depends on the global (not local) electric field, or depends on convection or electrodiffusion.

Indeed, in my opinion, when confronted with the models of polarization listed on the previous page, the classical Maxwell equations will be useful only when knowledge of the actual properties of polarization is not available. All the models listed involve time dependence in the polarization fields that are not included in the classical Maxwell equations as usually written.

## 5. Conclusions

A generalization of Maxwell’s P useful in a range of systems may emerge. The generalization would describe how the local electric field changes the distribution of charge, as one imagines that Maxwell hoped P and D would be.

Until then, one is left with:

(1) Bewilderingly complete measurements, over an enormous range of frequencies (e.g., [26,27,28,29,31,32,34,35]) of the dielectric properties and conductance of ionic solutions of varying composition and content. These measurements embarrass the theoretician with their diversity and complexity. They have not yet been captured in any formulas or programs less complicated than a look-up table of all the results.
(2) Computations of the motion of all charges on the atomic scale [21,88], described by the field equations of mechanics and electrodynamics [18].
(3) Reduced models. It is unlikely that the reduced models can be derived solely by mathematics. It is more likely that they must be ‘guessed and checked’ one by one, as most models are checked in science.

What should be done when little is known? Sadly, the actual properties of polarization are often unknown. Then, one is left with the over-approximated Equation (6) or nothing at all. It is almost never wise to assume polarization effects are negligible. Equation (6) is certainly better than nothing: Equation (6) can be particularly helpful if it is used gingerly: toy models can successfully represent an idealized view of a part of the real world of technological or biological importance, for example, electronic circuits or several properties of ion channels.

In some cases, the toy models can be enormously helpful. They allow the design of circuits in our analog and digital electronic technology [150,151,152,153]. They allow the understanding of selectivity [107,154,155,156] and current voltage relations of several important biological channel proteins in a wide range of solutions [107,157,158,159]. In other cases—for example, the description of ionic solutions with many components—the toy models can be too unrealistic to be useful. Experiments and experience can tell how useful the toy model actually is in a particular case: pure thought usually cannot.

## Data Availability

Not available.

## Appendix A P(x,y,z|t) in Proteins

Ambiguities in the meaning of the polarization field P(x,y,z|t) can cause serious difficulties in the understanding of protein function. Understanding protein function is greatly aided by knowledge of protein structure. The protein data bank contains 173,754 structures in atomic detail today (24 January 2021) and the number is growing rapidly as cryo-electron microscopy is used more and more.

Protein structures are usually analyzed with molecular dynamics programs that assume periodic boundary conditions and chemical equilibrium, i.e., no flows. Most proteins control large flows as part of their natural biological functions. Equilibrium hardly ever occurs in living biological systems. It seems obvious that equilibrium systems cannot provide general insight into flows, any more than a nonfunctional amplifier without a power supply can show how a functional amplifier works. Proteins are not periodic in their natural setting. It seems obvious that periodic systems with flow cannot conserve total current $J_{total}$ in general—or perhaps even in particular—as required by the Maxwell equations, see Equation (19). In other words, it is likely that molecular dynamics analyses of periodic structures do not satisfy the Maxwell equations, although almost all known physics does satisfy those equations.

It is also unlikely that standard programs of molecular dynamics compute electrodynamics of nonperiodic systems correctly, despite their use of Ewald sums, with various conventions, and force fields (tailored to fit macroscopic, not quantum mechanical) data. Compare the exhaustive methods used to validate results in computational electronics [61] with those in the computation of electric fields in proteins.

The electrostatic and electrodynamic properties of proteins are of great importance. Many of the atoms in a protein are assigned permanent charge greater than 0.2e in the force fields used in molecular dynamics, where e is the elementary charge, and these charges tend to cluster in locations most important for biological function, just as they cluster at high density near the electrodes of batteries and other electrochemical systems. Enormous densities of charge (>10 M, sometimes much larger) are found in and near channels of proteins [107,160,161,162] and in the ‘catalytic active sites’ [163] of enzymes. Such densities are also found near nucleic acids, DNA and (all types of) RNA and binding sites of proteins in general.

It seems likely that a hierarchy of models of different resolutions will be needed to compute the electrodynamics of proteins accurately enough to explain how the electrical properties of side chains (polarizability [21] and others) of a protein determine biological function. Analysis of gating currents suggests such an approach is feasible [17,18,20,21].
